# New Conditions for Obtaining the Exact Solutions of the General Riccati Equation

**DOI:** 10.1155/2014/401741

**Published:** 2014-08-18

**Authors:** Lazhar Bougoffa

**Affiliations:** Department of Mathematics, Faculty of Science, Al Imam Mohammad Ibn Saud Islamic University (IMSIU), P.O. Box 90950, Riyadh 11623, Saudi Arabia

## Abstract

We propose a direct method for solving the general Riccati equation *y*′ = *f*(*x*) + *g*(*x*)*y* + *h*(*x*)*y* ^2^. We first reduce it into an equivalent equation, and then we formulate the relations between the coefficients functions *f*(*x*), *g*(*x*), and *h*(*x*) of the equation to obtain an equivalent separable equation from which the previous equation can be solved in closed form. Several examples are presented to demonstrate the efficiency of this method.

## 1. Introduction

In realistic investigations of the dynamics of a physical system, nonlinearity may often be in the form of the well-known Riccati equation:

(1) $$\begin{array}{c} y^{\prime }=f\left(x\right)+g\left(x\right)y+h\left(x\right)y^{2}. \end{array}$$

The Riccati equation is used in different areas of physics, engineering, and mathematics such as quantum mechanics, thermodynamics, and control theory. It also appears in many engineering design simulations.

The first well-known result in the analysis of the Riccati equation is that [1, 2] if one particular solution *y*_1_ can be found to (1), then the general solution is obtained as

(2) $$\begin{array}{c} y=y_{1}+u, \end{array}$$

where *u* satisfies the corresponding Bernoulli equation

(3) $$\begin{array}{c} u^{\prime }-\left(g\left(x\right)+2h\left(x\right)y_{1}\right)u=h\left(x\right)u^{2}. \end{array}$$

The substitution that is needed to solve this Bernoulli equation is

(4) $$\begin{array}{c} z=\frac{1}{u}. \end{array}$$

A set of solutions to the Riccati equation is then given by

(5) $$\begin{array}{c} y=y_{1}+\frac{1}{z}. \end{array}$$

The second important result in the analysis of the solution of the Riccati equation is that the general equation can always be reduced to a second-order linear differential equation of the form [3, pages 23–25]

(6) $$\begin{array}{c} u^{\prime \prime }-P\left(x\right)u^{\prime }+Q\left(x\right)u=0, \end{array}$$

where

(7) $$\begin{array}{c} P\left(x\right)=g\left(x\right)+\frac{h^{\prime }\left(x\right)}{h\left(x\right)},\\ Q\left(x\right)=f\left(x\right)h\left(x\right). \end{array}$$

A solution of this equation will lead to a solution

(8) $$\begin{array}{c} y=-\frac{1}{h\left(x\right)}\frac{u^{\prime }}{u} \end{array}$$

of the original Riccati equation.

To our knowledge it is often very difficult, if not impossible, to find closed form solutions of such nonlinear differential equations. But a number of solutions of the Riccati equation can be obtained by assuming that the coefficients *f*(*x*), *g*(*x*), and *h*(*x*) satisfy certain constraints. Indeed, closed-form solutions are known if the following condition is satisfied [4]:

(9) f(x)+g(x)+h(x)=(log⁡α(x)β(x))′−α(x)−β(x)α(x)β(x)(α(x)h(x)−β(x)f(x)),

where *α*(*x*) and *β*(*x*) are arbitrary differentiable functions in *I* ⊂ *R* with *α*(*x*)*β*(*x*) > 0.

A new integrability condition for the Riccati equation was presented in [5] under the following constraint:

(10) $$\begin{array}{c} g\left(x\right)=\frac{F\left(x\right)-f\left(x\right)}{\int F\left(x\right)dx+C_{0}}-h\left(x\right)\left(\int F\left(x\right)dx+C_{0}\right), \end{array}$$

where *F*(*x*) is an arbitrary differentiable function on *I*.

Also, the general solution was found in [6] when

(11) $$\begin{array}{c} \frac{{\left(f(x)h(x)\right)}^{\prime }-2\left(g\left(x\right)+h^{\prime }\left(x\right)/h\left(x\right)\right)f\left(x\right)h\left(x\right)}{{\left(f(x)h(x)\right)}^{3/2}}=\pm \frac{4A}{\sqrt{B}} \end{array}$$

holds, where *A* and *B* are either constants or *A* = 0 and *B* is an arbitrary function.

In this paper, we present new solutions of the general Riccati equation. We first reduce it to an equivalent equation, and then we formulate the relations between the coefficients functions *f*(*x*), *g*(*x*), and *h*(*x*) of the equation to obtain the required relation

(12) $$\begin{array}{c} \frac{f^{\prime }\left(x\right)}{f\left(x\right)}-\frac{h^{\prime }\left(x\right)}{h\left(x\right)}-2g\left(x\right)=k\left(x\right)\sqrt{f\left(x\right)h\left(x\right)}, \end{array}$$

where *k*(*x*) is an arbitrary function. Therefore the given Riccati equation can be transformed into a separable equation, which can be easily solved in two cases: *k*(*x*) equals a constant *k*, or certain functions. Several examples are studied in detail to illustrate the proposed technique.

## 2. Exact Solutions of the Riccati Equation

We begin our approach by converting the general Riccati equation (1) to a simpler form by the substitution

(13) $$\begin{array}{c} u\left(x\right)=e^{-\int g(x)dx}y\left(x\right), \end{array}$$

which yields the equation

(14) $$\begin{array}{c} u^{\prime }=f_{0}\left(x\right)+f_{1}\left(x\right)u^{2}, \end{array}$$

where *f*_0_(*x*) = *e* ^−∫*g*(*x*)*dx*^ *f*(*x*) and *f*_1_(*x*) = *e* ^∫*g*(*x*)*dx*^ *h*(*x*).

Now, let

(15) $$\begin{array}{c} u=\sqrt{\left|\frac{f_{0}\left(x\right)}{f_{1}\left(x\right)}\right|}v, \end{array}$$

and consider the case *f*_0_(*x*)/*f*_1_(*x*) > 0. Thus the transformation of (15) to (14) yields

(16) $$\begin{array}{c} \left(\sqrt{\frac{f_{0}\left(x\right)}{f_{1}\left(x\right)}}\right)v^{\prime }+{\left(\sqrt{\frac{f_{0}(x)}{f_{1}(x)}}\right)}^{\prime }v=f_{0}\left(x\right)\left(1+v^{2}\right). \end{array}$$

Then (16) can be written in an equivalent form as

(17) $$\begin{array}{c} v^{\prime }=\sqrt{f_{0}\left(x\right)f_{1}\left(x\right)}\left(1+v^{2}\right)-{\left(\sqrt{\frac{f_{0}(x)}{f_{1}(x)}}\right)}^{\prime }\left(\sqrt{\frac{f_{1}\left(x\right)}{f_{0}\left(x\right)}}\right)v. \end{array}$$

An important remark can be made here. Equation (16) can be solved by assuming that the functions *f*_0_(*x*) and *f*_1_(*x*) satisfy the following condition:

(18) $$\begin{array}{c} {\left(\sqrt{\frac{f_{0}(x)}{f_{1}(x)}}\right)}^{\prime }\left(\sqrt{\frac{f_{1}\left(x\right)}{f_{0}\left(x\right)}}\right)=k_{1}\left(x\right)\sqrt{f_{0}\left(x\right)f_{1}\left(x\right)}, \end{array}$$

where *k*_1_(*x*) is an arbitrary function.

This condition can be written in an equivalent form as

(19) $$\begin{array}{c} \frac{f_{0}^{\prime }\left(x\right)}{f_{0}\left(x\right)}-\frac{f_{1}^{\prime }\left(x\right)}{f_{1}\left(x\right)}=k\left(x\right)\sqrt{f_{0}\left(x\right)f_{1}\left(x\right)},\;k\left(x\right)=2k_{1}\left(x\right), \end{array}$$

or equivalently

(20) $$\begin{array}{c} \frac{f^{\prime }\left(x\right)}{f\left(x\right)}-\frac{h^{\prime }\left(x\right)}{h\left(x\right)}-2g\left(x\right)=k\left(x\right)\sqrt{f\left(x\right)h\left(x\right)}. \end{array}$$

This leads to the following important cases that solve (16).

### 2.1. Case 1: *k*(*x*) Equals a Constant

Substituting *k*(*x*) = *k* into (19) gives

(21) $$\begin{array}{c} \frac{dv}{dx}=\sqrt{f\left(x\right)h\left(x\right)}\left(v^{2}-kv+1\right), \end{array}$$

which is a first-order separable differential equation, and we can obtain its closed form solution from

(22) $$\begin{array}{c} \int \frac{dv}{v^{2}-kv+1}=\int \sqrt{f\left(x\right)h\left(x\right)}dx. \end{array}$$

Based on the integral involving the rational algebraic functions of the form

(23) ∫dvav2+bv+c ={2tan−1((2av+b)/4ac−b2)4ac−b2, 4ac>b2,1b2−4aclog⁡|2av+b−b2−4ac2av+b+b2−4ac|, b2>4ac,1a(p−q)log⁡|v−pv−q|, b2>4ac,where  p  and  q  are  the  roots  of  av2+bv+c=0,−2tanh−1((2av+b)/b2−4ac)b2−4ac, b2>4ac,  (2av+b)2<b2−4ac,−2coth⁡−1((2av+b)/b2−4ac)b2−4ac, b2>4ac,  (2av+b)2>b2−4ac,−22av+b, b2=4ac,

in view of this, the solution in a closed form is given by

(24) v(x)={4−k22   ×tan(4−k22×[∫f(x)h(x)dx+C])+k2, 4>k2,(k+k2−4+(k2−4−k)×exp⁡[k2−4(∫f(x)h(x)dx+C)]) ×(1−exp⁡[k2−4(∫f(x)h(x)dx+C)])−1, k2>4,p+qexp⁡[(p−q)(∫f(x)h(x)dx+C)]1−exp⁡[(p−q)(∫f(x)h(x)dx+C)], k2>4,where  p  and  q  are  the  roots  of  v2−kv+1,k2−42tanh(−k2−42(∫f(x)h(x)dx+C))+k2, k2>4,  (2v−k)2<k2−4,k2−42coth⁡(−k2−42(∫f(x)h(x)dx+C))+k2, k2>4, (2v−k)2>k2−4,−1∫f(x)h(x)dx+C+k2, k2=4.

Once *v* is found then we can obtain *u* from (15).

Finally, after finding *u*, we can use *y* = *ue* ^∫*g*(*x*)*dx*^ to return to the original variable.

Our results can thus be summarized by the following lemma.

**Lemma 1 .**

If the coefficients of the general Riccati equation (1) satisfy the condition

(25) $$\begin{array}{c} \frac{f^{\prime }\left(x\right)}{f\left(x\right)}-\frac{h^{\prime }\left(x\right)}{h\left(x\right)}-2g\left(x\right)=k\sqrt{f\left(x\right)h\left(x\right)}, \end{array}$$

where *k* is a constant, then the general solutions of the Riccati equation can be exactly obtained as

(26) y(x)={f(x)h(x)4−k22 ×tan(4−k22[∫f(x)h(x)dx+C])+k2, 4>k2,f(x)h(x)(k+k2−4+(k2−4−k)×exp⁡[k2−4(∫f(x)h(x)dx+C)]) ×(1−exp⁡[k2−4(∫f(x)h(x)dx+C)])−1,   k2>4,f(x)h(x)p+qexp⁡[(p−q)(∫f(x)h(x)dx+C)]1−exp⁡[(p−q)(∫f(x)h(x)dx+C)], k2>4,where  p  and  q  are  the  roots  of  v2−kv+1,f(x)h(x)k2−42 ×tanh(−k2−42(∫f(x)h(x)dx+C))+k2, k2>4,  (2v−k)2<k2−4,e∫g(x)dxf(x)h(x)k2−42 ×coth⁡(−k2−42(∫f(x)h(x)dx+C))+k2, k2>4,  (2v−k)2>k2−4,−f(x)h(x)2∫f(x)h(x)dx+C+k2, k2=4,

where *C* is a constant.

**Remark 2 .**

For the case *f*_0_(*x*)/*f*_1_(*x*) < 0, proceeding as before, we obtain the following condition:

(27) $$\begin{array}{c} \frac{f^{\prime }\left(x\right)}{f\left(x\right)}-\frac{h^{\prime }\left(x\right)}{h\left(x\right)}-2g\left(x\right)=k\sqrt{-f\left(x\right)h\left(x\right)} \end{array}$$

and the first-order separable equation

(28) $$\begin{array}{c} \frac{dv}{dx}=\sqrt{-f\left(x\right)h\left(x\right)}\left(-v^{2}-kv+1\right). \end{array}$$

Thus the general solutions can be similarly found.

### 2.2. Case 2: *k*(*x*) Equals an Arbitrary Function

We have

(29) $$\begin{array}{c} \frac{dv}{dx}=\sqrt{f\left(x\right)h\left(x\right)}\left(v^{2}-k\left(x\right)v+1\right). \end{array}$$

This equation can be written in an equivalent form as

(30) $$\begin{array}{c} \frac{dv}{dx}=\sqrt{f\left(x\right)h\left(x\right)}\left[{\left(v-\frac{k\left(x\right)}{2}\right)}^{2}+\frac{4-k^{2}\left(x\right)}{4}\right]. \end{array}$$

The substitution of

(31) $$\begin{array}{c} w=v-\frac{k\left(x\right)}{2} \end{array}$$

into (30) yields

(32) $$\begin{array}{c} \frac{dw}{dx}=\sqrt{f\left(x\right)h\left(x\right)}\left[w^{2}+\frac{4-k^{2}\left(x\right)}{4}\right]-\frac{k^{\prime }\left(x\right)}{2}. \end{array}$$

If we assume that the function *k*(*x*) satisfies the following condition:

(33) $$\begin{array}{c} \sqrt{f\left(x\right)h\left(x\right)}\left[\frac{4-k^{2}\left(x\right)}{4}\right]-\frac{k^{\prime }\left(x\right)}{2}=\lambda \sqrt{f\left(x\right)h\left(x\right)}, \end{array}$$

where *λ* is a constant, then

(34) $$\begin{array}{c} \frac{k^{\prime }\left(x\right)}{k^{2}\left(x\right)-4+4\lambda }=-\frac{\sqrt{f\left(x\right)h\left(x\right)}}{2}, \end{array}$$

which is a separable equation. Thus

(35) k(x)={(2c11−λexp⁡[−21−λ∫f(x)h(x)dx]+21−λ)×(1−c1exp⁡[−21−λ∫f(x)h(x)dx])−1, 1−λ>0,2λ−1tan[−λ−1∫f(x)h(x)dx+c1λ−1], 1−λ<0,1(1/2)∫f(x)h(x)dx+c1, λ=1,

where *c*_1_ is an arbitrary integration constant. Then it is easy to solve (32) in closed form. Thus

(36) $$\begin{array}{c} \frac{dw}{dx}=\sqrt{f\left(x\right)h\left(x\right)}\left[w^{2}+\lambda \right], \end{array}$$

or

(37) $$\begin{array}{c} w=\sqrt{\lambda }\tan\left(\sqrt{\lambda }\int \sqrt{f\left(x\right)h\left(x\right)}dx+\sqrt{\lambda }c_{2}\right),\;\lambda \neq 0, \end{array}$$

where *c*_2_ is an arbitrary integration constant. Therefore,

(38) v=w+k(x)2=λtan(λ∫f(x)h(x)dx+λc2) +k(x)2, λ≠0.

Substituting the original expression for *y*, we obtain the final general solution as

(39) y(x)=f(x)h(x)[λtan(λ∫f(x)h(x)dx+λc2)+k(x)2], λ≠0.

For *λ* = 0, we have

(40) $$\begin{array}{c} w=\frac{-1}{\int \sqrt{f\left(x\right)h\left(x\right)}dx+c_{2}}. \end{array}$$

Thus

(41) $$\begin{array}{c} y\left(x\right)=\sqrt{\frac{f\left(x\right)}{h\left(x\right)}}\left[\frac{-1}{\int \sqrt{f\left(x\right)h\left(x\right)}dx+c_{2}}+\frac{k\left(x\right)}{2}\right]. \end{array}$$

We have the following.

**Lemma 3 .**

If the coefficients of the general Riccati equation (1) satisfy the condition

(42) $$\begin{array}{c} \frac{f^{\prime }\left(x\right)}{f\left(x\right)}-\frac{h^{\prime }\left(x\right)}{h\left(x\right)}-2g\left(x\right)=k\left(x\right)\sqrt{f\left(x\right)h\left(x\right)}, \end{array}$$

where *k*(*x*) is a function given by (35), then the general closed form solutions of the Riccati equation can be exactly obtained by (39) and (41).

## 3. Examples

Listed below are some special cases where the above conditions are satisfied and the general solutions of the Riccati equation are found.

### 3.1. The Coefficients *f*(*x*),   *g*(*x*), and *h*(*x*) Are Proportional

**Example 1 .**

Consider the following Riccati equation:

(43) $$\begin{array}{c} y^{\prime }=\varphi \left(x\right)\left(a+by+cy^{2}\right), \end{array}$$

where *f*(*x*), *g*(*x*), and *h*(*x*) are proportional; that is, *f*(*x*) = *aφ*(*x*), *g*(*x*) = *bφ*(*x*), and *h*(*x*) = *cφ*(*x*).

The condition equation (25) holds if and only if $k=-2b/\sqrt{\left|ac\right|}$. So the general solution to this equation can be found by

(44) y(x) ={|ac|4−k22 ×tan(4−k22[|ac|∫φ(x)dx+C])+k2, |ac|>b2,|ac|(k+k2−4+(k2−4−k)×exp⁡[k2−4(|ac|∫φ(x)dx+C)])×(1−exp⁡[k2−4(|ac|∫φ(x)dx+C)])−1, b2>|ac|,−|ac|2|ac|∫φ(x)dx+C+k2, b2=|ac|.

### 3.2. Special Case Where the Original Equation Has the Canonical Form

**Example 2 .**

Consider the following Riccati equation:

(45) $$\begin{array}{c} y^{\prime }=f\left(x\right)+y^{2}, \end{array}$$

where *f*(*x*) = 1/(*αx*+*β*)^2^, *g*(*x*) = 0, and *h*(*x*) = 1.

The condition equation (25) holds if and only if *k* = −2*α*. Thus, the general solution is given as

(46) y(x)={4−k221αx+β ×tan(4−k22[1αln⁡(αx+β)+C])+k2,1>α2,1αx+β(k+k2−4+(k2−4−k)×exp⁡[k2−4(1αln⁡(αx+β)+C)]) ×(1−exp⁡[k2−4(1α1αx+β+C)])−1, α2>1,−1αx+β2(1/α)ln⁡(αx+β)+C+k2, α2=1.

### 3.3. Equations Containing Power Functions

**Example 3 .**

Consider the following equation:

(47) $$\begin{array}{c} y^{\prime }=bx^{m}+ax^{n}y^{2}, \end{array}$$

where *f*(*x*) = *bx* ^*m*^, *g*(*x*) = 0, and *h*(*x*) = *ax* ^*n*^.

The condition equation (25) holds if and only if $k=\left(m-n\right)/\sqrt{\left|ab\right|}$ with *m* + *n* = −2. So the general solution to this equation can be found for any values of *a*, *b*, *m*, and *n* by

(48) y(x)={|ab|4−k22x(m−n)/2 ×tan(4−k22×[2m+n+2|ab|x(m+n+2)/2+C])+k2,4|ab|>(m−n)2,|ab|x(m−n)/2(k+k2−4+(k2−4−k)×exp⁡[k2−4×(2m+n+2×|ab|x(m+n+2)/2+C)]) ×(1−exp⁡[k2−4×(2m+n+2×|ab|x(m+n+2)/2+C)])−1,4|ab|<(m−n)2, −|ab|x(n−m)/2 ×2(2/(m+n+2))|ab|x(m+n+2)/2+C+k2, 4|ab|=(m−n)2.

**Example 4 .**

For the equation

(49) $$\begin{array}{c} y^{\prime }=cx^{-n-2}+\frac{b}{x}y+ax^{n}y^{2}, \end{array}$$

where *f*(*x*) = *ax* ^−*n*−2^, *g*(*x*) = *b*/*x*, and *h*(*x*) = *cx* ^*n*^, we get $k=-2(n+1+b)/\sqrt{\left|ac\right|}$, and the general solution to this equation is given by

(50) y(x)={|ac|4−k22x−(n+1) ×tan(4−k22[|ac|ln⁡x+C])+k2, |ac|>(n+1+b)2,|ac|x−(n+1)(k+k2−4+(k2−4−k)×exp⁡[k2−4(|ac|ln⁡x+C)]) ×(1−exp⁡[k2−4(|ac|ln⁡x+C)])−1, |ac|<(n+1+b)2,−|ac|x−(n+1)2|ac|ln⁡x+C+k2, |ac|=(n+1+b)2.

### 3.4. Other Equations

**Example 5 .**

Consider the following Riccati equation:

(51) $$\begin{array}{c} xy^{\prime }=x^{2m}+\left(m-n\right)y+x^{2n}y^{2}, \end{array}$$

where *f*(*x*) = *x* ^2*m*−1^, *g*(*x*) = (*m* − *n*)/*x*, and *h*(*x*) = *x* ^2*n*−1^. We get *k* = 0, and the general solution to this equation is given by

(52) y=f(x)h(x)4−k22 ×tan(4−k22(∫f(x)h(x)dx+C))+k2.

Thus the exact closed form solution is

(53) $$\begin{array}{c} y=x^{m-n}\tan\left(\frac{x^{n+m}}{n+m}+C\right), \end{array}$$

where *C* is a constant of integration.

**Example 6 .**

For the given equation

(54) $$\begin{array}{c} 2x^{2}y^{\prime }=-2a^{2}x+xy+2y^{2}, \end{array}$$

where *f*(*x*) = −*a* ^2^/*x*, *g*(*x*) = 1/2*x*, and *h*(*x*) = 1/*x* ^2^, we get *k* = 0, and the general solution to this equation is given by

(55) y=e∫g(x)dxf(x)h(x)4−k22 ×tan(4−k22(∫f(x)h(x)dx+C))+k2.

Thus the exact closed form solution is

(56) $$\begin{array}{c} y=\left|a\right|\sqrt{x}\tan\left(\frac{-2\left|a\right|}{x^{2}}+C\right), \end{array}$$

where *C* is a constant of integration.

### 3.5. Equations Containing Exponential Functions

**Example 7 .**

Consider the following Riccati equation:

(57) $$\begin{array}{c} y^{\prime }=be^{\mu x}+ay^{2}, \end{array}$$

where *f*(*x*) = *be* ^*μx*^, *g*(*x*) = 0, and *h*(*x*) = *a*.

The condition equation (42) holds if and only if $k(x)=\mu /\sqrt{\left|ab\right|}e^{-(\mu /2)x}$.

Combining this value of *k*(*x*) with (35), we get *μ* = 2. So $k(x)=\left(2/\sqrt{\left|ab\right|}\right)e^{-x}$. Thus the general solution to this equation can be found by (41); that is,

(58) $$\begin{array}{c} y\left(x\right)=\sqrt{\left|\frac{b}{a}\right|}\left[\frac{-1}{\sqrt{\left|ab\right|}e^{x}+c_{2}}+\frac{2}{\sqrt{\left|ab\right|}}e^{-x}\right]. \end{array}$$

## 4. Applications

### 4.1. The Central Potential Problem of the Power Law Type

Certain types of Newtons laws of motion are equivalent to the Riccati equation. For example, the equation for the energy conservation in the case of a central potential *V*(*r*) is given by the standard expression [7]

(59) $$\begin{array}{c} E=\frac{1}{2}m{\dot{r}}^{2}+\frac{l^{2}}{2mr^{2}}+V\left(r\right). \end{array}$$

Under the influence of a power law central potential *V*(*r*) = k*r ^*ϵ*^ and *E* = 0, where k* is the coupling constant and the exponent *ϵ* can be either positive or negative, (59) can be transformed into the Riccati equation

(60) $$\begin{array}{c} w^{\prime }=\frac{\epsilon +2}{2}+\frac{\epsilon +2}{2}w^{2}. \end{array}$$

A solution of this equation will lead to a solution

(61) $$\begin{array}{c} \frac{r^{\prime }}{r}=w \end{array}$$

of the original equation, where *r*′ = *dr*/*dθ* and *θ*(*t*) = 1/*mr* ^2^.

From the condition (25) of Lemma 1, we see that there exists a constant *k* = 0 for *ϵ* ≠ −2. Hence, the general solution of (60) is given by

(62) $$\begin{array}{c} w\left(\theta \right)=\tan\left(\frac{\left|\epsilon +2\right|}{2}\theta +C_{1}\right). \end{array}$$

Therefore,

(63) $$\begin{array}{c} r\left(\theta \right)=C_{2}{\left[\text{sec}\left(\frac{\left|\epsilon +2\right|}{2}\theta +C_{1}\right)\right]}^{2/\left|\epsilon +2\right|}, \end{array}$$

where *C*_2_ is a constant.

### 4.2. Damped Harmonic Oscillators

Various problems in quantum optics, superconductivity, and nonrelativistic quantum mechanics can be described classically by [8]

(64) $$\begin{array}{c} m\frac{d^{2}x}{dt^{2}}+\lambda \frac{dx}{dt}=-{\frac{\partial U}{\partial x}|}_{x=x(t)}, \end{array}$$

where *x*(*t*) is the particle coordinate, *m* is the mass of the particle, *λ* is a damping constant, and *U*(*x*, *t*) is a potential energy that accounts for the interaction of the particle with its environment. If *U*(*x*, *t*) = −*xμ*(*t*), where *μ*(*t*) is white or colored noise, then (60) becomes the well-known Langevin equation

(65) $$\begin{array}{c} m\frac{d^{2}x}{dt^{2}}+\lambda \frac{dx}{dt}-\mu \left(t\right)=0. \end{array}$$

Also the equation of motion is

(66) $$\begin{array}{c} \frac{d^{2}x}{dt^{2}}+2\beta \left(t\right)\frac{dx}{dt}+\omega^{2}\left(t\right)x=0, \end{array}$$

where *β* is the damping function and *ω* is the the natural frequency of the undamped oscillator.

The substitution *y* = *x*′/*x* converts (66) to the Riccati equation

(67) $$\begin{array}{c} y^{\prime }=-\omega^{2}\left(t\right)-2\beta \left(t\right)y-y^{2}. \end{array}$$

Here *f*(*t*) = −*ω* ^2^(*t*), *g*(*t*) = −2*β*(*t*), and *h*(*t*) = −1.

The condition (25) holds if and only if the coefficients *ω* and *β* satisfy the following condition:

(68) $$\begin{array}{c} \frac{\omega^{\prime }}{\omega }+2\beta =\frac{k}{2}\omega ,\;\text{where}\;k\;\text{is}\;\text{a}\;\text{constant}. \end{array}$$

Then (68) can be written in an equivalent form as

(69) $$\begin{array}{c} \omega^{\prime }=-2\beta \left(t\right)\omega +\frac{k}{2}\omega^{2}, \end{array}$$

which is a Bernoulli equation and can be readily solved for *ω* to obtain

(70) $$\begin{array}{c} \omega \left(t\right)=\frac{1}{-\left(k/2\right)+ce^{2\int \beta (t)dt}}. \end{array}$$

We conclude that if the coefficients *ω* and *β* satisfy (70), then the general solution *y*(*t*) of (67) is given by (26).

Returning to the original dependent variable by *y* = *x*′/*x*, we obtain the general solution to (66) as

(71) $$\begin{array}{c} x\left(t\right)=ce^{\int y(t)dt}, \end{array}$$

where *c* is a constant.

## 5. Conclusion

We have converted the Riccati equation into an equivalent equation; then, by using the integrability condition for this equation,

(72) $$\begin{array}{c} \frac{f^{\prime }\left(x\right)}{f\left(x\right)}-\frac{h^{\prime }\left(x\right)}{h\left(x\right)}-2g\left(x\right)=k\left(x\right)\sqrt{f\left(x\right)h\left(x\right)}, \end{array}$$

we obtain a separable equation. The first case *k*(*x*) = *k*, where constant *k* is a constant, is considered. Thus, the general solutions of the Riccati equation can be exactly obtained. The second integrability case is obtained for the reduced Riccati equation with an arbitrary function *k*(*x*).

We have considered several distinct examples to illustrate our new approach. The method is also applied to the Riccati equation arising in the solution of certain types of Newtons laws of motion and the damped harmonic oscillators equations.
